# Evaluating the performance of the MSKCC gastric cancer survival calculator in the Turkish population

**DOI:** 10.55730/1300-0144.5901

**Published:** 2024-07-04

**Authors:** İlknur DELİKTAŞ ONUR, Tuğba BAŞOĞLU, Nazım Can DEMİRCAN, Tuğba AKIN TELLİ, Rukiye ARIKAN, Özlem ERCELEP, Nazım Serdar TURHAL, Mehmet Akif ÖZTÜRK, Perran Fulden YUMUK, Faysal DANE

**Affiliations:** 1 Division of Oncology, Department of Internal Medicine, University of Health Sciences, Dr. Abdurrahman Yurtaslan Ankara Oncology Education and Research Hospital, Ankara, Turkiye; 2 Division of Oncology, Department of Internal Medicine, University of Health Sciences, Dr. Lütfi Kırdar Education and Research Hospital, İstanbul, Turkiye; 3 Division of Oncology, Department of Internal Medicine, University of Health Sciences, Çam and Sakura Education and Research Hospital, İstanbul, Turkiye; 4 Division of Oncology, Department of Internal Medicine, Memorial Şişli Hospital, İstanbul, Turkiye; 5 Division of Oncology, Department of Internal Medicine, Faculty of Medicine, Marmara University, İstanbul, Turkiye; 6 Division of Oncology, Department of Internal Medicine, Anadolu Medical Center, Kocaeli, Turkiye; 7 Division of Oncology, Department of Internal Medicine, Faculty of Medicine, Koç University, İstanbul, Turkiye; 8 Division of Oncology, Department of Internal Medicine, Altunizade Acıbadem Hospital, İstanbul, Turkiye

**Keywords:** MSKCC gastric tool, MSKCC gastric nomogram, prognostic tools, gastric cancer, gastric cancer prognostic tools

## Abstract

**Background/aim:**

The Memorial Sloan Kettering Cancer Center (MSKCC) nomogram was developed to predict survivorship in gastric cancer patients undergoing R0 resection. This study aimed to evaluate the predictive power of this nomogram in the Turkish patient population.

**Materials and methods:**

Gastric cancer patients over 18 years of age who were admitted to our clinic between 2000 and 2019 and underwent primary curative surgery and R0 resection were included in the study. The 5- and 9-year overall survival (OS) rates of 489 patients were analyzed. Real-life survival rates and those calculated using the MSKCC tool were compared in all the patients and subgroups. The relationship between the variables and survival were analyzed.

**Results:**

The 5-year median observed OS rate for all the patients was 51.7%, while the 5-year median OS rate calculated using the MSKCC tool was 48.5%. The difference between the expected and observed survival rates was 3.2%. The rates were similar and there was no statistically significant difference (p = 0.31). The 9-year median observed OS rate for all the patients was 41.4%, while the 5-year median OS rate calculated using the MSKCC tool was 41%. The difference between the expected and observed survival rates was 0.4%. The rates were similar and there was no statistically significant difference (p = 0.9).

**Conclusion:**

The 5- and 9-year survival rates estimated using the MSKCC tool were correlated with the 5- and 9-year survival rates in the real-life data. Hence, the use of the MSKCC prognostic tool in clinical practice should be expanded.

## 1. Introduction

Gastric cancer is the fifth leading cause of cancer worldwide [1]. It ranks fourth in cancer-related deaths worldwide [2]. Gastric cancer has a heterogeneous distribution in the world. It is most common in Asian societies, followed by South America and Eastern Europe [3]. The global incidence of gastric cancer has decreased over the last few decades [4,5]. It is thought that the widespread use of refrigerators contributed to this [6], as salt-based food storage methods have now decreased. In addition, bacterial and fungal contamination has also decreased [7]. In addition, *H. pylori* eradication is also thought to reduce the incidence of gastric cancer [8]. The most important prognostic factor in gastric cancer is the pathological stage of the tumor at the time of diagnosis. The most common staging system is the American Joint Committee on Cancer (AJCC) staging system. The staging schema of the AJCC/UICC is based on tumor, node, metastasis (TNM) classifications [9]. The TNM staging system provides information about stage-specific prognosis. It also ignores disease-specific variables that affect recurrence and survival. For example, it does not include demographic characteristics such as age, or pathological characteristics such as perineural invasion. Although the AJCC/TNM cancer staging system has become more complex with many modifications, there are still aspects of the AJCC stages that are insufficient [10]. One of the important reasons for this is that the system depends more on anatomy than multidisciplinary measurements [11]. However, survival rates show heterogeneity even among patients in the same stage. This led to the idea that the development of individualized prognosis predictive models would be useful to clinicians in deciding on treatment strategies and follow-up protocols. Based on this idea, calculators have been created to help estimate survival and recurrence rates [12].

The Memorial Sloan Kettering Cancer Center (MSKCC) gastric cancer nomogram is a survival prediction tool developed by Kattan et al. [12]. It was created using the variables of age, sex, depth of tumor invasion, number of positive lymph nodes, number of negative lymph nodes, Lauren histology, tumor location, and tumor size. The nomogram gives the estimated percentage of the 5- and 9-year survival rates after these data are entered. The nomogram was developed in 2003 and was developed by including patients between 1985 and 2002. At that time, the effectiveness of adjuvant and perioperative treatments in gastric cancer had not yet been demonstrated, and patients who underwent upfront surgery and R0 resection were included in the study [12].

The aim of the current study was to compare the 5- and 9-year survival outcomes estimated using the MSKCC survival calculator with the real-life outcomes of the patients with stage I–III gastric cancer.

## 2. Materials and methods

**Figure 1:**
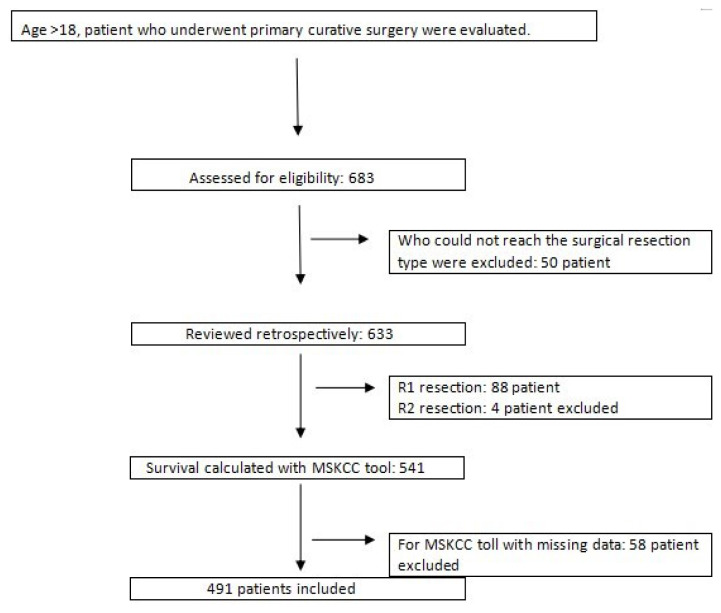
Inclusion and exclusion criteria.

The data of the patients were obtained from the hospital registry system. Patients with gastric cancer aged 18 years or older who underwent primary curative surgery with R0 resection were included in the study. Fifty patients whose surgical resection type was not available, 88 who underwent R1 resection, and four who underwent R2 resection were excluded from the study. Fifty-eight patients who did not have sufficient data for the MSKCC survival calculator were excluded from the study (Figure 1).

## 3. Statistical analysis

All the analyses were performed using IBM SPSS Statistics for Windows 23.0 (IBM Corp., Armonk, NY, USA). The primary endpoint was determined as the overall survival (OS). The observed survival rates of the patients were calculated using the Kaplan–Meier method in all the patient groups and subgroups. Median and standard error values of the observed survival times were obtained. The percentage of patients alive at month 60 was recorded as the 5-year OS rate, and that of patients alive at month 108 was recorded as the 9-year OS rate.

The relationship between the variables and survival rates was examined via univariate analyses. The variables determined as significant in the univariate analysis were evaluated via multivariate analysis with the Cox-regression model. Survival rates in the subgroups were compared using the log-rank test. p < 0.05 was considered statistically significant.

For each patient, the estimated 5- and 9-year OS rates were calculated using the MSKCC tool. The observed survival rates for all the patients and patient subgroups were compared with the calculated survival rates. This comparison was performed with the online program.^1^ MedCalc uses the N-1 chi-squared test as recommended by Campbell [13] and Richardson [14]. The confidence interval was calculated according to the recommended method given by Altman et al. [15]. p < 0.05 was considered statistically significant.

## 4. Results

The clinicopathologic characteristics are given in Table 1. Over 29% (n = 145) of the patients were female and 70.5% (n = 347) were male. The mean age at diagnosis was 59.2 years. Twenty-three of the patients received perioperative chemotherapy and 352 received adjuvant chemotherapy. Since the follow-up of 101 patients for adjuvant chemotherapy was carried out at an external center, the treatment information they received could not be clearly accessed. The median follow-up period for all the patients was 66 months, while the median follow-up period for the living patients was 55 months.

Patients aged 65 years or older were assigned to the older group, while those under the age of 65 were assigned to the younger group. A strong relationship was found between age and OS. The median OS for patients under the age of 65 was 89.9 months, while that for those over the age of 65 was 39.5 months (p = 0.0001) (Table 2).

**Figure 2:**
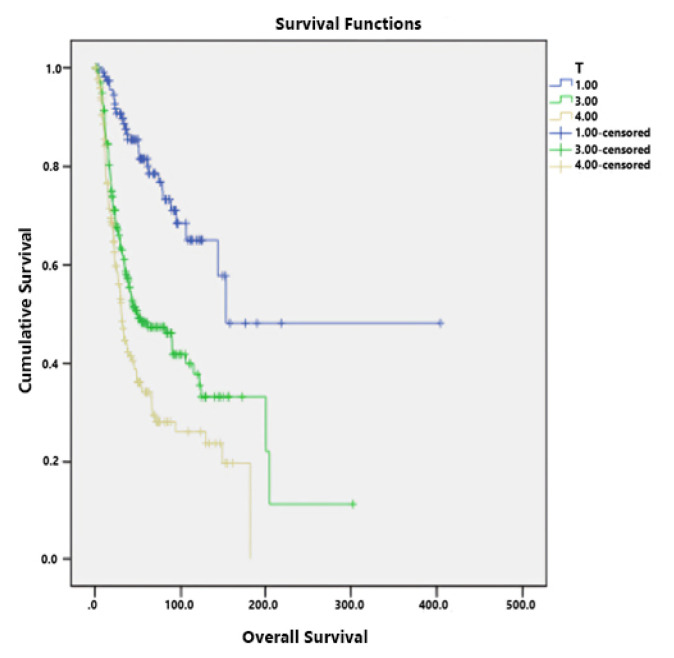
Relationship between the T stage and OS.

**Figure 3:**
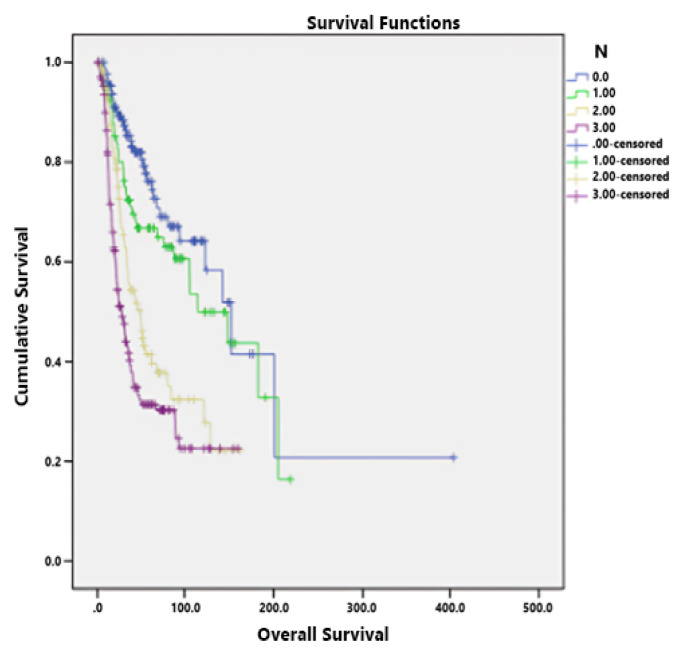
Relationship between the N stage and OS.

The T4 stage had a negative effect on survival (HR: 2.33, 95% CI: 1.16–4.66, p = 0.017) (Figure 2). Likewise, the negative effect of the N3 stage on survival was statistically significant (HR: 2.14, 95% CI: 1.16–3.94, p = 0.014) (Figure 3). The presence of vascular invasion also had a statistically significant effect on survival (HR: 1.66, 95% CI: 1.1–2.51, p = 0.015) (Table 2).

Real-life 5- and 9-year OS results were compared with the MSKCC estimated 5- and 9-year OS results. The 5-year median observed OS rate in all the patients was 51.7%, while the 5-year median OS rate calculated using the MSKCC tool was 48.5%. The difference between the expected and observed survival rates was 3.2%. The rates were similar and there was no statistically significant difference (p = 0.31). (Table 3).

The expected and observed 5-year OS rates were also compared for the variable subgroups. The 5-year expected and observed survival rates were generally similar in the patient subgroups, although there were statistically significant differences in some of the subgroups. The observed 5-year survival rate in the younger patient group was 57.8%, while the expected survival rate using the MSKCC tool was 45%. The MSKCC tool calculated a 5-year OS rate that was 12.8% lower than the observed survival rate in the younger patient group. This difference was statistically significant (p = 0.0011). The observed 5-year survival rate in the older patient group was 39.5%, while the expected rate was 51%. The MSKCC tool estimated a 5-year OS rate that was 11.5% higher than the observed OS rate in the older patient group. This difference was statistically significant (p = 0.0361). The survival rates calculated using the nomogram for the T4, N3, and diffuse subtypes were lower than those observed in real life.

The observed 9-year median OS rate in all the patients was 41.4%, while the observed 5-year median OS rate calculated using the MSKCC tool was 41%. The difference between the expected and observed survival rates was 0.4%. The rates were similar and there was no statistically significant difference (p = 0.9). (Table 4).

The expected and observed 9-year OS rates were also compared in terms of the variable subgroups. While no significant difference was detected in most of the variable subgroups, there was a statistically significant difference in the age group. The observed 9-year survival rate in the younger patient group was 46.9%, while the expected survival rate using the MSKCC tool was 38%. The 9-year OS rate was 8.9% lower than the observed survival rate in younger patient group. This difference was statistically significant (p = 0.02). The observed 9-year survival rate in the older patient group was 30.1% and the expected rate was 44%. The observed 9-year OS rate was 13.9% higher than that in the older patient group. This difference was statistically significant (p = 0.009). Similar to the 5-year survival, the estimated survival rate was lower for the T4, N3, and diffuse subtypes.

## 5. Discussion

The predictive power of the MSKCC survival calculator for 5- and 9-year survival was quite strong. The prediction of survival in the whole patient group overlapped with the real-life data. Validation studies of the MSKCC nomogram have been conducted in various countries. Its effectiveness in the European population was evaluated for the first time in a study conducted by Novotny et al. [16], which included 862 stage I–III gastric cancer patients who were operated on and underwent R0 resection between 1985 and 2003. The patients did not receive postoperative adjuvant therapy [16]. In their study, Kattan et al. [12] revealed that the age at diagnosis, primary site, number of positive nodes, and depth of invasion were significantly associated with disease-specific survival in the Cox model. In a study by Novotny et al. [16], age at diagnosis, primary site, number of positive nodes, and depth of invasion had a significant impact on disease-specific survival of the patients. Additionally, the number of negative nodes removed, and the presence of postoperative complications were identified as independent predictors of disease-specific survival. Similarly, in the present study, age at diagnosis, T stage, and N stage were independent factors for OS, while the presence of vascular invasion was also an independent factor (Table 2). Predictions from the nomogram were compared with those in the study of Novotny et al. [16], which were obtained using the AJCC stage groupings. They found that nomogram discrimination was superior to that of the AJCC stage groupings (concordance index: 0.770 vs. 0.756, p = 0.008).

In a study by Chen et al. [17], the validation of the MSKCC nomogram in the Chinese population was evaluated. They included 979 stage I–III gastric cancer patients who were operated on and underwent R0 resection between 1985 and 2007. Some of the patients received adjuvant chemotherapy treatment. The effectiveness of the MSKCC nomogram in predicting survival was compared with the AJCC staging system, and although some of the patients received adjuvant treatment, the MSKCC nomogram was superior to the AJCC system.

A study published by Zhou et al. [18] was conducted in a single center in China, which included 150 patients who were resected at stage I–III R0 and received postoperative chemoradiation. Its effectiveness was evaluated using the MSKCC nomogram. In the current study, the survival predicted by the nomogram was approximately 20% less than the observed one. Researchers have argued that the group underestimated by the MSKCC nomogram is the one that truly benefits from chemoradiotherapy. They hypothesized that this nomogram could be developed for use in the selection of the patient group that would benefit from chemoradiation [18].

In their study, Akgül et al. [19] compared the effectiveness of the MSKCC and Asia nomograms in 145 stage I–III gastric cancer patients who were operated on and underwent R0 resection between 2006 and 2013. The MSKCC nomogram was superior to the Asia nomogram in predicting survival. It was not stated whether the patients included in this study received adjuvant treatment.

When all the patients were evaluated together, the 5- and 9-year survival rates predicted using the MSKCC survival calculator coincided with the real-life data. However, when the patient subgroups were evaluated, several differences were observed. According to Lauren histology, survival was predicted to be lower in the diffuse and mixed types. Likewise, while it predicted lower 5- and 9-year survival for T4 tumors and N3 disease, it predicted higher 9-year survival for T2–3 stages and N0 disease. It also predicted a lower 5-year survival in the lymph node-positive and perineural invasion subgroups. The patients included the study by Kattan et al. [12], when they created the MSKCC calculator, did not receive adjuvant therapy. In the present study, the majority of the patients received adjuvant treatment. We believe that the lower survival prediction by the calculator in groups with poor prognostic features was due to the fact that most of the patients received adjuvant treatment. Zhou et al. [18], in their study, also argued that the subgroup predicted to be lower using the MSKCC nomogram consisted of patients who benefited from adjuvant chemoradiotherapy, but they conducted a subgroup analysis in their own time. In the current study, insight was gained regarding patients who benefited from the treatment through subgroup analysis.

The MSKCC survival calculator predicted lower 5- and 9-year survival rates in the younger patient group, while in the older patient group, it predicted higher rates. As information was not available on the comorbidities of the patients in the study, it was unknown whether the patients died from the disease or from other causes. We believe that the difference between the predicted and observed survival in the younger and older patient groups may have been due to older patients dying from comorbidities. Kattan et al. [12] also stated that one of the weaknesses of the calculator is that it does not include comorbidities.

In conclusion, despite the advances in treatment options and increased access to treatment, gastric cancer continues to constitute a significant percentage of cancer-related mortality and morbidity. Even at the same stage, there are different outcomes in terms of response to treatments, survival, and recurrence rates. To determine the treatment scheme, to inform the clinician about the treatment options and efficacy of the therapies, and to assist the clinician in the follow-up process, there is a need for prognostic and predictive calculating tools created with the variables we have knowledge about. The effectiveness of the MSKCC survival calculator in predicting the 5- and 9-year OS is superior to the survival prediction of the AJCC classification, as shown in the Chinese and German studies. In Türkiye, the survival data predicted using the MSKCC nomogram and real-life data in the Turkish population were similar. We believe that larger multicenter studies are needed so that the MSKCC nomogram can be used more widely in the clinical setting, in selecting patients who will benefit from adjuvant therapy and in predicting patient survival.

In the current study, the survival rates calculated by the nomogram and observed in real life in the entire population and patient subgroups were compared. One of the main limitations of the study was that the median follow-up period was 66 months. We believe that the follow-up period should be extended in order to evaluate the 9-year survival estimates more accurately. Other limitations of the study were that it was retrospective and that the adjuvant treatment protocols received by the patients were varied.

## 6. Conclusion

The MSKCC prognostic tool can be used to predict survival in clinical practice in gastric cancer patients who underwent surgery and R0 resection. We believe that an updated nomogram can be developed to identify patients who will benefit from adjuvant and neoadjuvant treatment in gastric cancer patients, and our multicenter study on this subject continues.

## Figures and Tables

**Table 1:** Clinicopathologic characteristics of patients.

Variables	Cases	(%)
**Sex**		
**Male**	347	70.5
**Female**	145	29.5
**Age**		
**Mean (range)**	59.2 (24–89)	
**Primary site**		
**Gastroesophageal junction**	12	3
**Cardia**	89	18.1
**Fundus**	11	2.2
**Corpus**	168	34.1
**Antrum**	185	37.6
**Prepyloric**	20	4.1
**Others**	4	0.8
**Lauren classificatio**n		
**Intestinal**	279	56.7
**Diffuse**	159	32.3
**Mixed**	53	10.8
**Tumor size (cm)**		
**Mean**	5.65	
**T stage**		
**T0**	2	0.4
**T1**	37	7.6
**T2**	76	15.6
**T3**	199	40.9
**T4A**	160	32.9
**T4b**	12	2.5
**Lymph node stage**		
**N0**	133	27.4
**N1**	82	16.9
**N2**	99	20.4
**N3a**	116	23.9
**N3b**	56	11.5
**Type of surgery**		
**Total gastrectomy**	247	50.2
**Distal subtotal gastrectomy**	196	39.9
**Proximal subtotal gastrectomy**	33	6.7

**Table 2:** Univariate and multivariate analyzes of factors related to OS.

Factor	Univariate Analysis	Multivariate Analysis	
	*p*	HR (%95 CI)	*p*
Age (<65 vs. ≥65)	0.0001		
Tumor location	0.012		
Lauren classification	0.05		
T stage	0.0001	2.33 (1.16–4.66)	0.017
N stage	0.0001	2.14 (1.16–3.94)	0.014
LNR	0.0001		
Lymphatic invasion	0.0001		
Vascular invasion	0.0001	1.66 (1.10–2.51)	0.015
Perineural invasion	0.0001		
Grade	0.0001		

LNR: lymph node ratio.

**Table 3:** Comparison of estimated 5-year survival rates using the MSKCC tool with real-life data.

	5-year observed		5-year estimated				
	N	5-year survival percent	N	5-year survival percent	Difference	95% CI	p
Age							
<65	324	57.8	327	45	12.8	5.12 to 20.26	0.0011
≥65	165	39.5	165	51	11.5	0.76 to 21.86	0.0361
Sex							
Female	144	53.1	145	57	3.9	−7.48 to 15.14	0.5
Male	345	51	347	45	6.0	−1.43 to 13.34	0.11
Tumor location							
1	104	44.7	104	36	8.7	−4.47 to 21.55	0.2
2	176	48.4	179	45	3.4	−6.91 to 13.61	0.5
3	205	59.6	205	57	2.6	−6.89 to 12.02	0.6
Lauren classification							
Intestinal	278	5.8	279	59	2.2	−5.96 to 10.32	0.6
Diffuse + mixed	210	45.2	212	32	13.2	3.91 to 22.17	0.005
T stage							
0-1-2	116	81.5	116	87	5.5	−3.97 to 14.93	0.25
3	200	48.3	202	47.5	0.8	−8.88 to 10.46	0.8
4	173	34.2	174	20	14.2	4.85 to 23.23	0.003
N stage							
0	132	76.1	133	83	6.9	−2.84 to 16.52	0.16
1	82	66.7	83	64	2.7	−11.64	0.7
2	101	41.5	101	40	1.5	−11.85	0.8
3	173	31.4	174	15.5	15.9	7.01 to 24.5	0.005
LN dissection type							
D0–1	174	51	176	54	3	−7.39 to 13.3	0.57
D2–3	308	52.1	309	43	9.1	1.22 to 16.82	0.02
Lymphatic invasion							
+	85	77.4	85	85	7.6	−4.24 to 19.26	0.2
−	343	45	344	36	9	1.65 to 16.2	0.016
Vascular invasion							
+	193	66.5	193	68	1.5	−7.81 to 10.77	0.75
−	247	38.4	248	33	5.4	−3.02 to 13.74	0.21
Perineural invasion							
+	165	67.4	166	63.5	0.6	−4.25 to 15.3	0.9
−	270	42.1	272	32	10.1	1.96 to 18.04	0.015
Grade							
1 + 2	165	64.1	166	63.5	0.6	−9.6 to 10.84	0.9
3 + undifferentiated	233	44.1	234	36	8.1	−0.78 to 16.8	0.07
All patients	489	51.7	492	48.5	3.2	−3.04 to 9.41	0.31

**Table 4:** Comparison of estimated 9-year survival rates using the MSKCC tool with real-life data.

	9-year observed		9-year estimated			
	N	9-year survival percent	N	9-year survival percent	Difference	p
Age						
<65	324	46.9	327	36	8.9	0.02
≥65	165	30.1	165	44	13.9	0.009
Sex						
Female	144	50.5	145	50	0.5	0.9
Male	345	37.5	347	37	0.5	0.9
Tumor location						
1	104	32	104	29	3	0.63
2	176	42.4	179	37	5.4	0.29
3	205	47.3	205	50	2.7	0.58
Lauren classification						
Intestinal	278	43.5	279	52	8.5	0.044
Diffuse + mixed	210	38	212	24	14	0.0019
T stage						
0-1-2	116	65	116	84	19	0.0009
3	200	39.9	202	39.5	0.4	0.9
4	173	26.2	174	14	12.2	0.0046
N stage						
0	132	64.2	133	79	14.8	0.0077
1	82	53.5	83	58	4.5	0.5
2	101	32.4	101	32	0.4	0.9
3	173	22.6	174	10	12.6	0.0046
LN dissection type						
D0–1	174	39.7	176	47	7.3	0.17
D2–3	308	42.6	309	34	8.6	0.02
Lymphatic invasion						
+	85	60.3	85	82	21.7	0.002
−	343	35.3	344	28.5	6.8	0.056
Vascular invasion						
+	193	51.8	193	62	10.2	0.04
−	247	31.1	248	26	5.1	0.2
Perineural invasion						
+	165	55.3	166	68	12.7	0.018
−	270	30	272	24	6	0.1
Grade						
1 + 2	165	51.9	166	56.5	4.6	0.4
3 + undifferentiated	233	33.8	234	28	5.8	0.17
All patients	489	41.4	492	41	0.4	0.9

## Footnotes

1 MedCalc Software Ltd (2024). Easy-to-use statistical software [online]. Website https://www.medcalc.org/calc/comparison_of_proportions.php [accessed 20 January 2020].
